# Recent Advances in Phenazine Natural Products: Chemical Structures and Biological Activities

**DOI:** 10.3390/molecules29194771

**Published:** 2024-10-09

**Authors:** Wei Huang, Yupeng Wan, Shuo Zhang, Chaozhi Wang, Zhe Zhang, Huai Su, Peng Xiong, Feifei Hou

**Affiliations:** 1 School of Life Sciences and Medicine, Shandong University of Technology, Zibo 255000, China; huangwei2205@163.com (W.H.); chaozhi1216@126.com (C.W.); 2 Shandong Freda Biotech Co., Ltd., Jinan 250101, China; suhuai@biofreda.com; 3 CAS Key Lab of Biobased Materials, Qingdao Institute of Bioenergy and Bioprocess Technology, Chinese Academy of Sciences, Qingdao 266101, China; wanyp@qibebt.ac.cn (Y.W.); zhangzhe@sdut.edu.cn (Z.Z.); 4 College of Traditional Chinese Medicine, Shandong University of Traditional Chinese Medicine, Jinan 250355, China; zhangtcm@163.com

**Keywords:** phenazine natural products, structural diversity, biological activities, biosynthesis

## Abstract

Phenazine natural products are a class of colored nitrogen-containing heterocycles produced by various microorganisms mainly originating from marine and terrestrial sources. The tricyclic ring molecules show various chemical structures and the decorating groups dedicate extensive pharmacological activities, including antimicrobial, anticancer, antiparasitic, anti-inflammatory, and insecticidal. These secondary metabolites provide natural materials for screening and developing medicinal compounds in the field of medicine and agriculture due to biological activities. The review presents a systematic summary of the literature on natural phenazines in the past decade, including over 150 compounds, such as hydroxylated, *O*-methylated, *N*-methylated, *N*-oxide, terpenoid, halogenated, glycosylated phenazines, saphenic acid derivatives, and other phenazine derivatives, along with their characterized antimicrobial and anticancer activities. This review may provide guidance for the investigation of phenazines in the future.

## 1. Introduction

**Figure 1:**
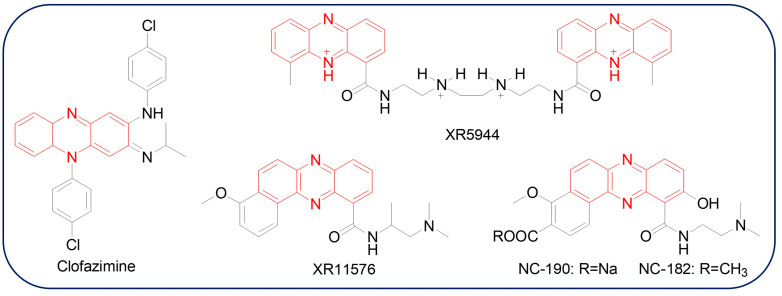
The chemical structures of clofazimine, XR5944, XR11576, NC-190, and NC-182.

**Figure 2:**
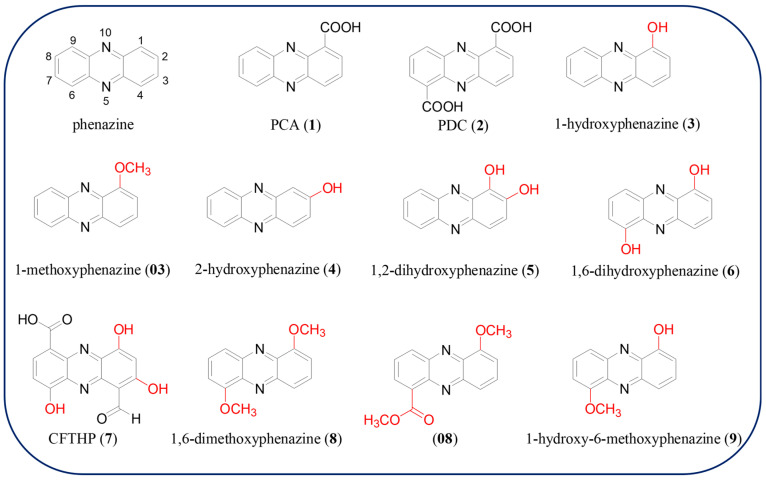
The chemical structures of compounds phenazine, **1**–**8**, **03**, and **08**.

Natural products are considered to be important sources of drug discovery. As the technologies for isolation, purification, and detection developed, great interest has been shown in underexplored natural products [1,2]. The first phenazine product, “pyocyanine” (now known as pyocyanin, PYO), was determined as a blue pigment from purulent wounds of patients in 1859; however, its structure was not established until nearly 100 years later [3]. Since PYO was identified as a *Pseudomonas aeruginosa* metabolite, more than 6000 kinds of phenazines have been found, of which there are over 100 phenazine natural products [4,5,6,7]. The skeleton structure of phenazine derivatives is a pyrazine ring (1,4-diazabenzene) coupling two annulated benzenes. The physical and chemical properties of phenazines depend on the types and positions of different substituent functional groups. Their antibiotic, antifungal, antiparasitic, antimalarial, insecticidal, and antitumor biological activities are presumed to be derived from the toxic reactive oxygen species (ROS) formation ability, the facilitation of energy generation, involvement in iron homeostasis via Fe (III) reduction, participation as signal molecules via the activation of the Fe-containing transcription factor SoxR, DNA Π–Π interaction and intercalation, and biofilm morphogenesis [8]. Some natural phenazines might be candidates or prodrugs not only for both bacteria and fungi but also as anticancer or antitumor agents. For example, Clofazimine or Lamprene™, which is a riminophenazine compound, has been successfully applied in clinics for the treatment of leprosy and tuberculosis due to antimicrobial activity and immunosuppressive properties [9]. Fused aryl phenazine derivatives XR11576, XR5944, NC-182, and NC-190 show significant anticancer activity, and some of them are also under clinical studies (Figure 1) [10]. One of the natural phenazine products, phenazine-1-carboxylic acid (PCA, **1**), has been developed as a commercialized biopesticide “Shenqinmycin” in China since 2011 and is widely used to treat fungal diseases in crops (Figure 2) [11,12]. Natural phenazines are produced directly from various microbes, such as *Pseudomonas* spp., *Streptomyces* spp., *Vibrio* spp., *Burkholderia* spp., *Brevibacterium* spp., *Pseudonocardia* spp., and archaeal *Methanosarcina* spp., etc. [13,14]. The biological synthesis of phenazines has been extensively studied for a long time due to their beneficial interactions with plants and animals. Chorismate, an intermediate of the shikimate pathway, is currently known as the common phenazine biosynthetic precursor. The *phz*-operon, which has been proven to spread in all phenazine-producing Gram-negative and Gram-positive bacteria by horizontal gene transfer [15,16], codes five conserved enzymes: ketosteroid isomerase (PhzB), isochorismatase (PhzD), anthranilate synthase (PhzE), diaminopimelate epimerase similar protein (PhzF), and FMN-dependent enzyme (PhzG). These *phz*-operon enzymes could convert chorismic acid into PCA and/or phenazine-1,6-dicarboxylic acid (PDC), which are catalyzed to create more complex structures by modification enzymes (proteins for hydroxylation, decarboxylation, methylation, *N*-oxidation, glycosylation, and prenylation, *etc*.). The progress regarding biosynthesis and metabolic engineering research of phenazines in the past decades has been summarized in other reviews [13,17,18].

Over the past decade, research reports on natural phenazines isolated and structurally identified have been constantly growing, and phenazines have increasingly attracted people’s attention in the field of medicine and agriculture. This review presents a comprehensive and methodical complication of the chemical structures of phenazines (hydroxylated, *O*-methylated, *N*-methylated, *N*-oxide, terpenoid, halogenated, glycosylated phenazines, saphenic acid derivatives, and other phenazine derivatives), along with their associated biological activities and pharmacological effectiveness. The aim of this work is to elucidate the structure and function to find suitable applications of phenazines.

## 2. Structural Diversities and Biological Activities

### 2.1. Basic Phenazine Structures

Phenazine heterocycles contain fused benzene moieties at the carbon positions of a pyrazine nucleus (Figure 2). Within the scope of existing knowledge, all phenazine molecules are derived from two basic carboxylate-bearing phenazines, PCA (**1**) and PDC (**2**) (Figure 2). In recent years, the bioactivity of PCA and PDC has been further evaluated. PCA has been isolated from a large number of microbes, such as *Streptomyces*, *Pseudomonas*, *Lysobacter*, *Xenorhabdus*, *Truncatella*, *Paenibacillus*, *Rhodococcus*, *Klebsiella*, *Kitasatospora*, *Burkholderia*, and *Bacillus*, etc. It shows broad-spectrum antifungal activity against a series of phytopathogens like *Phytophthora apsica*, *Gibberella zeae*, *Verticillium dahlia*, *Phaeoacremonium minimum*, *Fomitiporia mediterranea*, *Neofusicoccum parvum*, *Sclerotinia sclerotiorum*, *Phyricularia grisea*, *Streptomyces scabies*, and *Rhizoctonia solani* [19,20,21,22,23,24,25,26]. Based on its outstanding biological activities, PCA was developed as a new registered biopesticide, Shenqinmycin, in 2011 by Xu and colleagues [11,12]. At present, it is widely used in China as a key fungicide to prevent rice sheath blight, pepper blight, downy mildew, leaf spot, fusarium head blight, canker, etc. [27]. PDC, with two carboxyl groups at 1, 6-position of the phenazine ring is commonly isolated from *Streptomyces* spp., *Erwinia herbicola*, *Pseudomonas* spp., and *Lysobacter antibioticus*. It has lethal activity against Gram-negative (e.g., *E. coli*) and Gram-positive (e.g., *Bacillus subtilis*) bacteria at a concentration of 37 mM [11,28,29,30,31,32]. In addition, PDC shows cytotoxicity against MCF7, HeLa, and HT29 cells of breast, cervical, and prostate cancer [30].

### 2.2. Phenazines with Hydroxyl and Methoxyl Moieties

**Figure 3:**
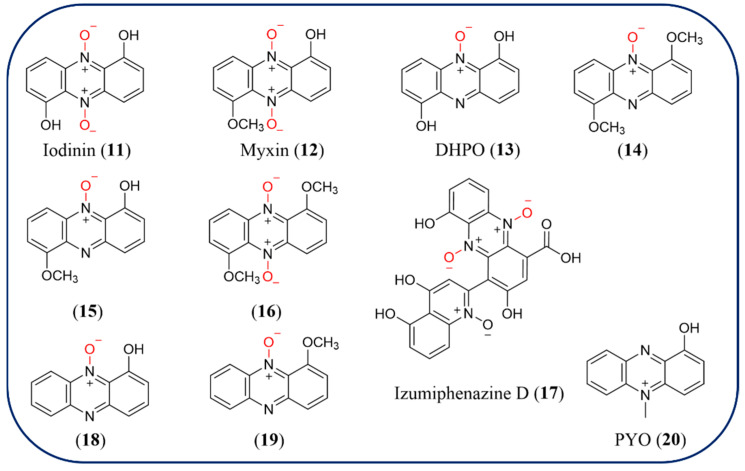
The chemical structures of *N*-oxide and *N*-methylated phenazines.

The two most common types of post-modification in natural phenazines are hydroxylation and hydroxy-methylation. Ten hydroxylated and *O*-methylated phenazines (**3**–**10**, **03**, **08**) are summarized in this review (Figure 2). 1-Hydroxyphenazine (**3**) and 2-hydroxyphenazine (**4**) with a single hydroxyl, 1,2-dihydroxyphenazine (**5**), and 1,6-dihydroxyphenazine (**6**) carry two hydroxyl groups at different positions, and 1-carboxyl-6-formyl-4,7,9-trihydroxy-phenazine (CFTHP, **7**) bearing three hydroxyl groups at 4,7,9 position have all been reported as phenazine hydroxylation products [33]. They are mostly present in *Streptomyces* spp., *Pseudomonas* spp., *Rhodococcus* spp., *Sphingomonas wittichii*, *Lysobacter antibioticus*, and *Xenorhabdus szentirmaii*, and can inhibit *Aeromonas hydrophila*, *Fusarium oxysporum*, *Rhizoctonia solani*, *Pythiumultimum*, *Alternaria alternata*, *Alternaria solani*, *Colletotrichum acutatum*, *Curvularia andropogonis*, *Fusarium moniliforme*, *Pythium aphanidermatum*, and tumor cells [34,35,36,37,38,39]. Compound **4** was reported to possess a better inhibitory effect on the take-all disease of wheat than PCA [40]. Two *O*-methylated phenazines compounds 1,6-dimethoxyphenazine (**8**) and 1-hydroxy-6-methoxyphenazine (**9**) were isolated from *Lysobacter antibioticus* OH13 with weak antibacterial activity [39]. Further, 1-methoxyphenazine (**03**) and methyl-6-methoxyphenazine-1-carboxylate (**08**) were isolated from *Streptomyces luteireticuli* NIIST-D75, and both compounds were shown explicitly in elaborate in vitro experiments as potentiators of ciprofloxacin for antibacterial activity with highly reduced dosage [41]. Strepphenazine A (**10**), which contains hydroxyl, carboxyl, and methoxyl groups, has been detected in the culture broth of *Streptomyces* strain YIM PH20095, showing antifungal activity against *Fusarium oxysporum*, *Plectosphaerella cucumerina*, *Alternaria panax*, and *Phoma herbarum*, which prevented root-rot disease in *Panax notoginseng* with minimal inhibitory concentrations of 16–32 μg/mL [42]. This study shows that *Streptomyces* strain YIM PH20095 provides new sources for the development of biological control agents to prevent the infection of pathogenic fungi of *P. notoginseng*. Although *O*-methylated natural products are very common, the *O*-methylation is important for the antibiotic activity of phenazines. For example, compound **03** has much higher antifungal activity against *Bipolaris maydis*, *Alternaria solani*, and *Aspergillus flavus* than compound **3** [43]. Besides that, the antimicrobial activity of myxin (**12**) is also much higher than iodinin (**11**) (Figure 3) [44].

### 2.3. N-Oxide Phenazines

Molecules with *N*-oxide can be found in various natural sources including plants, microorganisms, and animals. The *N*-oxide group may provide water solubility or decrease membrane permeability or immunogenicity and has a special redox reactivity. The level of *N*-oxide functionality is essential for cytotoxicity. These features render the phenazine *N*-oxides particularly valuable as starting points for antitumor agents, which selectively promote apoptotic cell death at low levels of oxygen saturation [45,46]. Nine natural phenazine *N*-oxides (**11**–**19**) are described in this paper (Figure 3). Compound iodinin (1,6-dihydroxyphenazine-5,10-dioxide, **11**), which has been identified in *Streptosporangium* sp. DSM 45942, and *Lysobacter antibioticus* OH13, is the *N*5, *N*10-dioxide of 1,6-dihydroxyphenazine [47,48]. The compound demonstrates high antimicrobial and cytotoxic activity and is particularly potent against acute myeloid leukemia, acute promyelocytic leukemia cells, and other leukemia cells, with EC50 values for cell death up to 40 times lower for leukemia cells when compared with normal cells. The results of experiments show that **11** represents a promising structure in the development of anticancer therapy. Another well-known compound is myxin (1-hydroxy-6-methoxyphenazine-*N*5, *N*10-dioxide, **12**) isolated from *Lysobacter antibioticus*, which has significant antimicrobial activity. DNA intercalation, the production of reactive oxygen species (ROS) and inhibition of topoisomerases and metal chelation are proposed action modes for the biological activity of iodinin and myxin [49]. Other phenazine oxides produced by *Lysobacter antibioticus*, including 1,6-dihydroxyphenazine*-N*5-oxide (DHPO, **13**), 1,6-dimethoxyphenazine-*N*5-oxide (**14**), 1-hydroxy-6-methoxyphenazine-*N*5-oxide (**15**), and 1,6-dimethoxyphenazine-*N*5,*N*10-dioxide (**16**) [39]. Izumiphenazine D (**17**), characterized by *Streptomyces* sp. IFM 11204, is the first example of quinoline-*N*-oxide containing phenazine natural product. Compound **17** has a synergistic effect in combination with TRAIL against AGS cells, and does not exhibit inhibition of Wnt signal transcription activity even at 50 μM [50]. Recently, two novel phenazine *N*-oxides, 1-hydroxyphenazine-*N*10-oxide (**18**) and 1-methoxyphenazine-*N*10-oxide (**19**) were artificially designed and biosynthesized in *Pseudomonas chlororaphis* [43,51]. 1-methoxyphenazine-*N*10-oxide (**19**) shows better activity than other phenazine products against plant pathogenic fungi, *Bipolaris maydis*, *Alternaria solani*, *Fusarium graminearum*, *Rhizoctonia solani*, and *Aspergillus flavus*. NaphzNO1 from *Nocardiopsis* sp. 13-12-13 and LaPhzNO1 from *L. antibioticus* OH13 are responsible for adding an oxygen atom to the nitrogen atom in phenazine *N*-oxides biosynthesis, they are all NADPH-dependent, flavin-containing *N*-monooxygenases. NaphzNO1 catalyzes the formation of **18** from **3**; however, it is unable to convert **03** into **19**. Furthermore, LaPhzNO1 is able to convert **9** into **15** but not **12** and cannot convert **8** to any of the *N*-oxide products in the in vitro assays. These results demonstrate that once the neighboring hydroxyl groups at the C1 or C6 locations are methylated, the *N*-monooxygenase cannot oxidize the phenazine nitrogen atom and may provide clues on new phenazine derivatives biosynthesis [39].

### 2.4. Phenazines with N-Methyl Moiety

There are two *N*-methylated phenazine natural products, pyocyanin (PYO, **20**) and 5-methyl phenazine-1-carboxylic acid (5MPCA) (Figure 3). Moreover, 90–95% of *Pseudomonas aeruginosa* produce one of the most famous phenazines PYO, a redox-active compound that has been heterologously expressed in *E. coli* [52]. It shows potential against bacterial and fungal strains and has many damaging effects on mammalian cells because of its ability to generate oxidative stress [53,54,55]. It is a fascinating molecule in many fields such as medicine, agriculture, aquaculture, and biosensors [5,56]. The precursor of PYO, 5MPCA, was also characterized as a secondary metabolite from *Pseudomonas*. 5MPCA exhibits selective cytotoxicity towards lung (A549) and breast (MDA MB-231) cancer cell lines in a dose-dependent manner, with an IC50 value of 488.7 ± 2.52 nM and 458.6 ± 2.48 nM, respectively [57]. It exhibits inhibition of cell viability and DNA synthesis and induces G1 cell-cycle arrest and apoptosis in cancer cells through the mitochondrial intrinsic pathway via the activation of caspase-3 and downregulation of Bcl-2 protein.

### 2.5. Phenazines with Carboxamide Moiety

**Figure 4:**
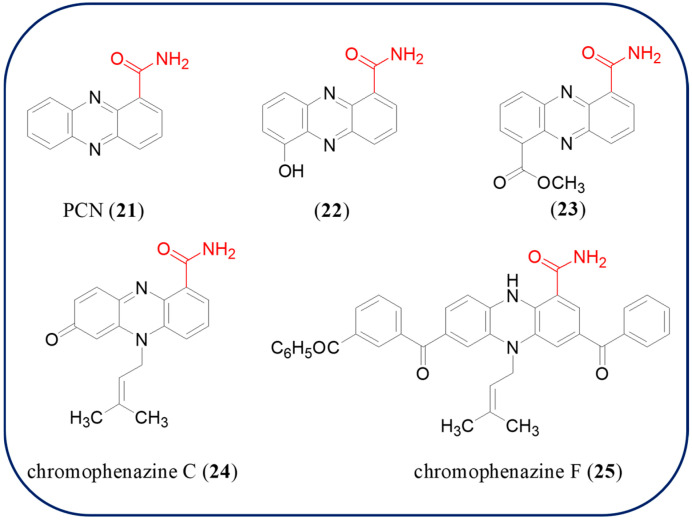
The chemical structures of compounds **21**–**25**.

Compounds **21**–**25** are carboxamidated phenazines described in this review (Figure 4). Phenazine-1-carboxamide (PCN, **21**) is mainly isolated from *Pseudomonas*, *Streptomycetes*, *Nigrospora oryzae*, and *Pantoea agglomerans* [58,59,60]. PCN exhibits antagonistic activities against a wide range of plant pathogenic fungi *Fusarium oxysporum* f. sp. *niveum* (causing fusarium wilt), *Fusarium graminearum* (causing fusarium head blight), and *Rhizoctonia solani* (causing rice sheath blight), etc. [61,62], as well as inhibitory effect on different cancer cell lines A549, HeLa, and SW480 between the concentration of 32 and 40 μM [63]. There was research that showed that PCN secreted by a bacterium *Pseudomonas piscium* ZJU60 from the wheat head directly manipulates the activity of the plant pathogenic fungus *Fusarium graminearum* histone acetyltransferase domain of FgGcn5 and suppresses fungal virulence, growth, and mycotoxin biosynthesis [64,65]. As a result, it provided a unique example that an antagonistic microbe could inhibit the virulence and growth of a plant pathogenic fungus by affecting its histone modification. PCA amide is a promising skeleton for designing more potent phenazine-based fungicides. 6-Hydroxyphenazine-1-carboxamide (**22**), methyl 6-carbamoylphenazine-1-carboxylate (**23**), chromophenazine C (**24**), and chromophenazine F (**25**) are four novel phenazine derivatives with carboxamide moiety obtained from *Streptomycetes* [66,67]. Compounds **22** and **23** exhibit moderate antifungal and antibacterial activities against *Fusarium oxysporum* (ATCC 7808), *Fusarium solani* (ATCC 36031), *Staphylococcus aureus* (ATCC 25923), and *Staphylococcus albus* (ATCC 10231), respectively [67]. However, the novel complicated compounds **24** and **25** did not show any activity against *B. subtilis*, *E. coli*, *M. miehei*, *S. aureus*, and *C. albicans* [66]. Further investigation on the bioactivity of compounds **22**–**25** needs to be undertaken in the future.

### 2.6. Terpenoid Phenazines

**Figure 5:**
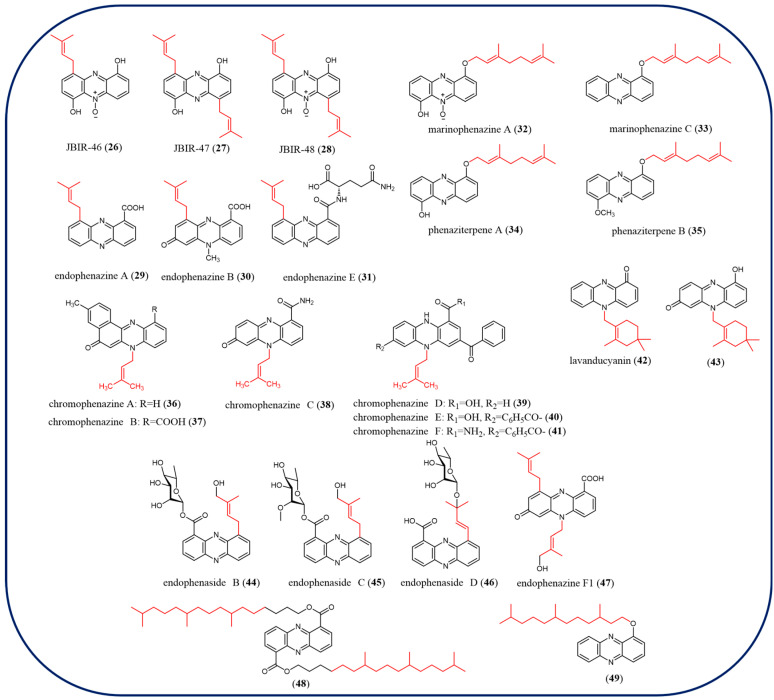
The chemical structures of terpenoid phenazines.

Terpenoid phenazines are a large class of compounds and contain common structural features of isoprenylated *C*, *N*, and *O* side chains or complex structures. In this review, 24 terpenoid phenazines (**26**–**49**) are summarized (Figure 5). However, most terpenoid phenazines exhibit moderate or weak antibacterial activity. A series of prenylated phenazine compounds have been discovered in *Streptomyces*. For instance, *C*-prenylated phenazines, including JBIR-46 (**26**), JBIR-47 (**27**), JBIR-48 (**28**), endophenazine A (**29**), endophenazine B (**30**), and a novel phenazine, endophenazine E (**31**), which represents a glutamine *α*-amino attached to the carboxyl group of endophenazine A. Compounds **29**, **30**, and **31** show antimicrobial activity against the Gram-positive *Bacillus subtilis* and Gram-negative *E. coli* [68]. Compounds **32**–**35** are *O*-prenylated phenazines and phenazines **36**–**43** are *N*-prenylated derivatives [69,70,71]. According to the result of the test, marinophenazine A/C (**32**–**33**), phenaziterpene A (**34**), and phenaziterpene B (**35**) had no discernable biological activity [72]. Only chromophenazine D (**42**) demonstrated moderate effectiveness against bacteria among the six *N*-prenylated phenazines, chromophenazines A–F (**36**–**41**) [66]. Lavanducyanin (**42**) and its congener 1-hydroxy-7-oxolavanducyanin (**43**) isolated from *Streptomyces* show intriguing cytotoxic activities [73]. Antibacterial assays revealed that **43** had significant but reduced anti-Gram-positive bacterial activity compared with **42**, and both compounds were all inactive against Gram-negative bacteria [73]. Endophenaside B (**44**), endophenaside C (**45**), endophenaside D (**46**), and endophenazine F1 (**47**), which possess substantial antimicrobial activities, were isolated from *Kitasatospora* sp. [68]. Compounds **48** and **49**, with terpenoid aliphatic chains at positions 1 and/or 6 of the phenazine ring, were identified in the *Pseudomonas aeruginosa* strain RRLJ 04 culture [74]. The strain exhibited growth promotion and disease control in pigeon peas, both compounds **48** and **49** showed in vitro antibiosis against *Fusarium udum* and can be further exploited for growth enhancement and disease control of other crops.

### 2.7. Glycosylated Phenazines

**Figure 6:**
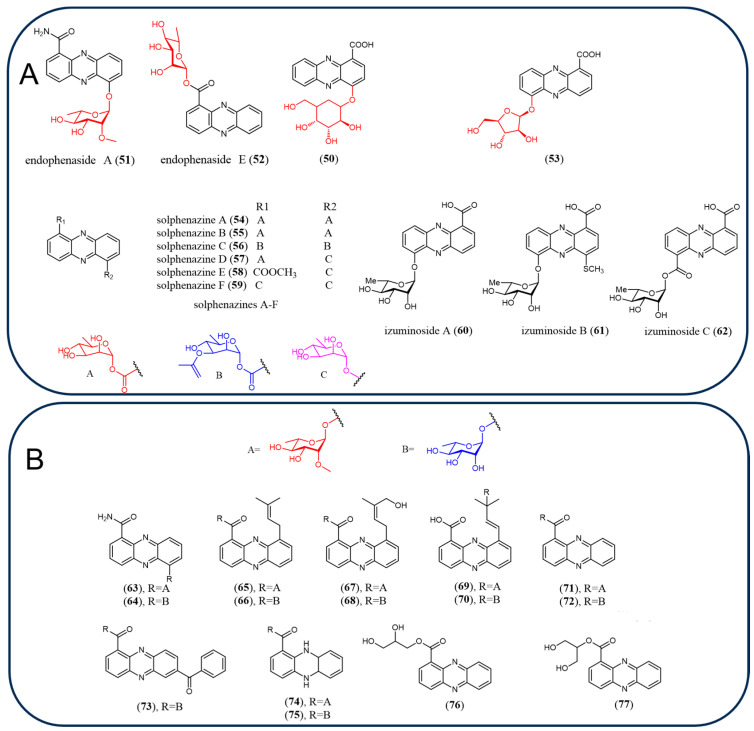
The chemical structures of glycosylated phenazines: (**A**) novel glycosylated phenazine natural products. (**B**) 2′-*O*-methylated/2′-*O*-unmethylated rhamnose glycosylated phenazines and two tautomeric glycerides naturally occurring phenazines.

The increasing bacterial multi-drug resistance necessitates novel drug-discovery efforts. One way of obtaining novel chemistry is the decoration of molecules by glycosylation because the pharmacological properties of the parent scaffold can be dramatically influenced by glycosylation, such as in anthracycline, avermectin, aureolic acid, and enediyne antibiotics [75]. Glycosylation is a common modification of natural phenazine products with high diversity in both the sugar moieties and the targeted aglycones. A total of 28 glycosylated phenazines (**50**–**77**) and their activities are described in this literature (Figure 6A,B). Compound 4-*O*-glucosyl-1-carboxyl-phenazine (**50**) from *Streptomyces* sp. strain UICC B-92 exhibited inhibitory potency against Gram-positive bacteria *B. cereus* strain ATCC 10876 and *S. aureus* strain ATCC 25923 [76]. A number of novel glycosylated phenazine-type antibiotic endophenasides A–E (**51, 44, 45, 46, 52**) were produced by *Kitasatospora* sp. MBT66 [68]. Endophenaside A (**51**) and endophenaside C (**45**) are phenazines that contain a rare 2′-*O*-methylation of the sugar moiety. Endophenasides A–E showed antimicrobial activity against the Gram-positive *Bacillus subtilis* or Gram-negative *E. coli* [68]. *Nannocystis pusilla* strain Ari7 has been demonstrated to produce 1,6-dihydroxyphenazine glycosylated derivative, 1-hydroxyphenazine-6-yl-*α*-*D*-arabinofuranoside (**53**) [77]. Novel glycosylated phenazine products solphenazines A–F (**54**–**59**), along with izuminosides A–C (**60**–**62**), were isolated from *Streptomyces* sp. strain DL-93 and IFM 11260, respectively. Compounds **54**–**59** did not show any antifungal or antibacterial activity; however, **54**, **55,** and **59** displayed some cytotoxicity against HCT-116 cancer cells (Figure 6A), the cytotoxicity was not associated with DNA intercalations and topoisomerase inhibition and the mechanisms of action were uncertain [78]. Compounds **61** (10 μM) and **62** (60 μM) in combination with TRAIL showed synergistic activity in sensitizing TRAIL-resistance AGS cells. 2′-*O*-methylated/2′-*O*-unmethylated rhamnose glycosylated phenazines and two tautomeric glyceride naturally occurring phenazine products (**63**–**77**) were characterized in *Kitasatospora* sp. MBT66 fermentation broth (Figure 6B) [79]. As described above, most glycosylated phenazine natural products do not have remarkable activity, and the activities of glycosylated phenazines need to be further investigated.

Besides that, unprecedented 5*N*-glucosylated phenazine derivatives 7-imino-5*N*-(1′β-D-glucopyranosyl)-5,7-dihydrophenazine1-carboxylic acid and 3-imino-5*N*-(1′β-D-glucopyranosyl)-3,5-dihydrophenazine-1-carboxylic acid were purified and identified when *Bacillus* sp. G2112 and *Pseudomonas* sp. G124 were co-cultivated. And 3-imino-5*N*-(1′β-D-glucopyranosyl)-3,5-dihydrophenazine-1-carboxylic acid did not inhibit *Bacillus* sp. G2112 which proved that the observed modification constitutes a resistance mechanism [80]. *Caenorhabditis elegans*, which is a useful model organism to study the xenobiotic detoxification pathways, can detoxify **3** by adding one, two, or three glucose molecules in N2 worms [81]. The structures of the trisaccharide sugar phenazines made by *C. elegans* were characterized, and the results showed that one of the sugar modifications contains an *N*-acetylglucosamine (GlcNAc) in place of glucose [82]. Xenobiotic detoxification can weaken the potential of the producing organism or pesticides against pathogens and should be considered during the development of biocontrol strategies.

### 2.8. Halogenated Phenazines

**Figure 7:**
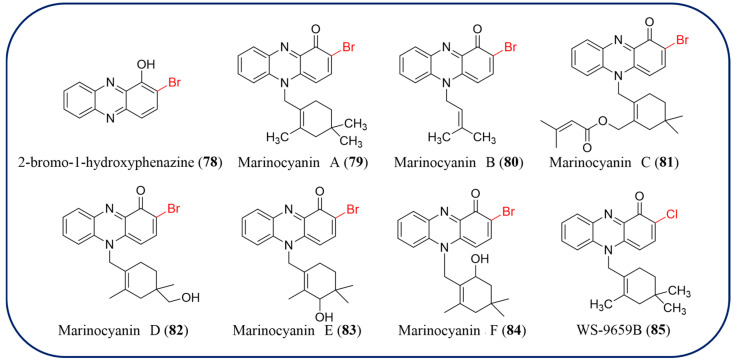
The chemical structures of halogenated phenazine natural products.

Halogenated phenazine natural products are rare in nature, and their biosynthetic pathways have not been discovered. The halogenated phenazines are 2-bromo-1-hydroxyphenazine (**78**) from *Streptomyces* sp. CNS284 showed strong cancer cell cytotoxicity (IC_50_ = 0.1 μM against HCT-116) and weak to moderate activity in the NF-κB-luciferase assay (IC_50_ = 73 μM) [83]. Six brominated phenazines, marinocyanins A–F (**79**–**84**), that have strong to weak cytotoxicity against HCT-116 human colon carcinoma and possess modest antimicrobial activities against *Staphylococcus aureus* and *Candida albicans* were obtained from *Streptomyces* with the lavanducyanin chlorinated analog WS-9659B (**85**) (Figure 7) [84]. Up to now, there have been no reports on fluorinated or iodinated phenazine natural products. But a novel series of halogenated phenazines has been developed by chemical synthesis methods due to their potent antibacterial and biofilm eradication activities against critical Gram-positive pathogens, including *Staphylococcus aureus*, *Staphylococcus epidermidis*, and *Enterococcus faecium* [85,86]. These compounds have the potential to dramatically impact future therapies related to biofilm-associated infections.

### 2.9. Saphenic Acid Derivatives

**Figure 8:**
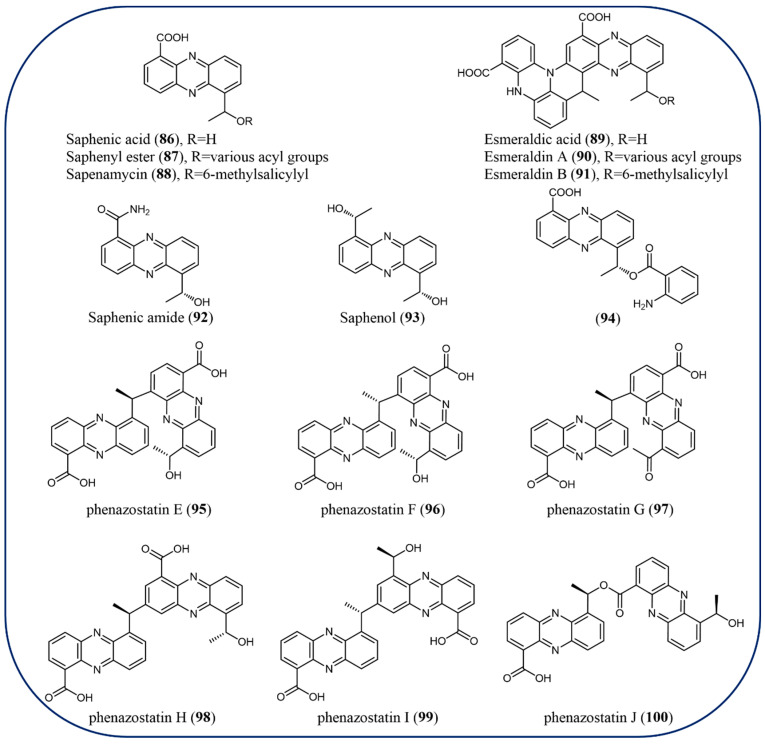
The chemical structures of saphenic acid derivatives.

Microorganisms are a rich source of structurally unique bioactive metabolites and drug candidates. Saphenic acid (6-(1-hydroxyethyl) phenazine-1-carboxylic acid, **86**) isolated from *Streptomyces*, is a common pharmacophore for many antibiotics and antitumor reagents including DC-86-M and phenazostatins [87]. Moreover, its derivatives such as saphenyl ester (**87**), saphenamycin (**88**), esmeraldic acid (**89**), esmeraldin A (**90**), and esmeraldin B (**91**) are present in the metabolites of *Streptomyces* [87]. In particular, marine microbes from the deep sea are a relatively untapped reservoir of metabolites with structural and biological diversity waiting to be discovered. For example, saphenic amide (**92**), saphenol (**93**), 6-[1-(2-aminobenzoyloxy) ethyl]-1-phenazinecarboxylic acid (**94**), phenazostatins E-J (**95**–**100**) were isolated from the culture broth of marine yeast-like fungus *Cystobasidium laryngis* derived from deep-sea sediments of the Indian Ocean Ridge (Figure 8) [31,88,89]. Compounds **92** and **93** showed a nitric oxide (NO) production-inhibitory effect against lipopolysaccharide (LPS)-induced murine macrophage RAW 264.7 cells without cytotoxicity at concentrations up to 30 µg/mL. Moreover, diphenazine phenazostatin J (**100**) exhibited significant antineuroinflammatory activity with an IC_50_ value of 0.30 μM and cytotoxicity against the NUGC-3 (stomach) cell line with an IC_50_ value of 7.7 nM, exhibiting 19-fold stronger activity than adriamycin. Interestingly, **100** demonstrated stronger cytotoxicity than its oxidized form (*R*)-6-(1-((6-acetylphenazine-1-carbonyl)oxy)ethyl) phenazine-1-carboxylic acid and positive control, indicating that the ester linkage and the hydroxyethyl group attached to the phenazine scaffold are essential for the activities [88]. Compound **100** could be a potential agent for the development of antineuroinflammatory and anticancer leads, and the underlying mechanisms for the biological activities and the structure–activity relationship are needed for further study.

### 2.10. Phenazines with Sulfur

**Figure 9:**
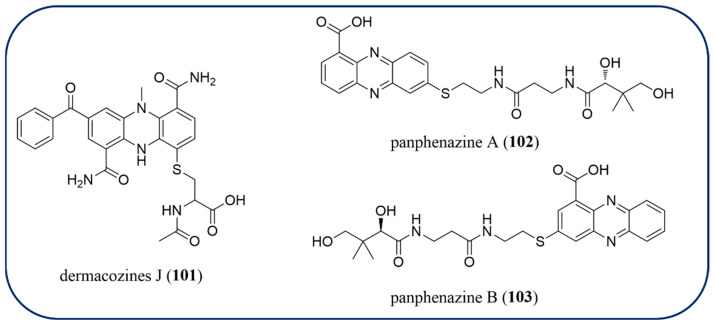
The chemical structures of phenazines with sulfur.

The four phenazines with sulfur (**61**, **101**–**103**) are introduced in this review (Figure 9). Izuminoside B (**61**), the phenazine with a C-4 coupled methylsulfanyl group at the *para*-position to carboxylic acid has been obtained from *Streptomyces* sp. IFM 11260 [90]. The activity of compound **61** has been described in Section 2.7. Compound dermacozines J (**101**) isolated from *Dermacoccus abyssi* has been established to have an *N*-acetylcysteine moiety attached to C-9, and it demonstrated radical scavenging activity [91]. Panphenazine A (**102**) and B (**103**) were characterized by actinomycete *Kitasatospora* sp. HKI 714 as unusual pantetheine-containing phenazines, which were proposed to be synthesized by radical-induced conjugate addition [92]. This study indicated that phenazines possess the ability to capture biogenic thiols, crosslink proteins, and, ultimately, contribute to protein degeneration/aggregation processes.

### 2.11. Other Phenazine Derivatives with Special Structures

**Figure 10:**
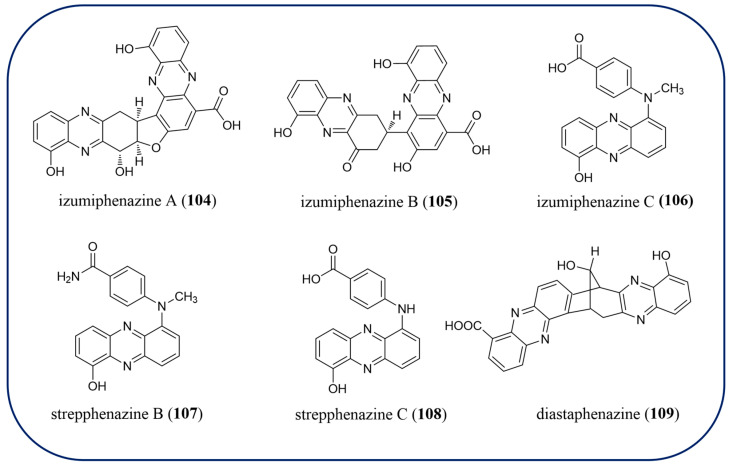
The chemical structures of izumiphenazines and analogs.

**Figure 11:**
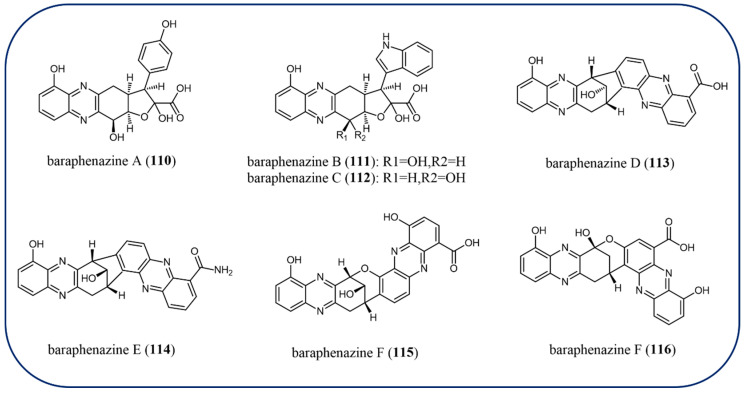
The chemical structures of baraphenazines.

**Figure 12:**
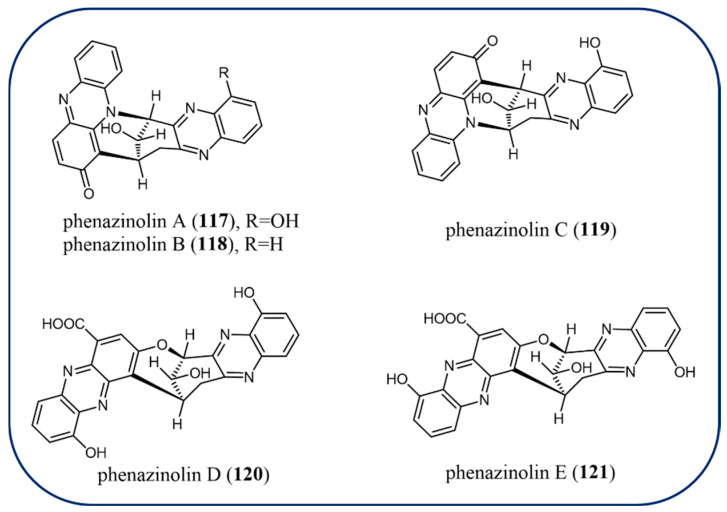
The chemical structures of phenazinolins.

Recently, phenazine natural products with a wider range of structural variations (**104**–**150**) have been identified from microorganisms. Red solids phenazines izumiphenazines A–C (**104**–**106**) were isolated from *Streptomyces*, and izumiphenazine A (**104)** is the first instance of tetrahydrofuran ring connected phenazine dimer [93]. Compounds **104**–**106** were evaluated to have a synergistic effect in combination with TRAIL (TNF-related apoptosis-inducing ligand) against AGS cells. Izumiphenazine C (**106)** analogs strepphenazine B–C (**107**–**108**) have been characterized by *Streptomyces* with antifungal activities [42]. A novel dimeric phenazine, diastaphenazine (**109)**, was also obtained from the fermentation broth of *Streptomyces* (Figure 10). Compound **109** has antibacterial activity against *S. aureus* but is inert against *E. coli* and *C. albicans*, according to the antimicrobial test [94]. Among the seven new phenazine-based metabolites—baraphenazines A–G (**110**–**116**) (Figure 11)—compounds **104**–**106** represent the first reported examples of fused 5-hydroxyquinoxaline/alpha-keto acid-based moiety [95]. Furthermore, baraphenazines D (**113)** and E (**114)** present two new diastaphenazine-type C-C-fused phenazine-based congeners, while baraphenazines F (**115)** and G (**116)** are two phenazinolin-type C-O-fused compounds. Only baraphenazines E (**114**) exhibited significant activity in antibacterial and fungal tests at the doses studied. A tin mine tailings-derived *Streptomyces* was found to create a novel family of diphenazines known as phenazinolins A–E (**117**–**121**). Phenazinolins A–C (**117**–**119**) exhibited amazing violet color and antagonistic activity for *Bacillus subtilis*, *Staphylococcus aureus*, *Aspergillus niger*, and *Botrytis cinerea* with MIC values in the range of 12–27 μM. The IC_50_ values of compounds **117**–**119** in vitro cytotoxicity against GLC, P388, XWLC, and H460 human cancer cell lines were in the range of 14–40 μM (Figure 12) [96,97].

**Figure 13:**
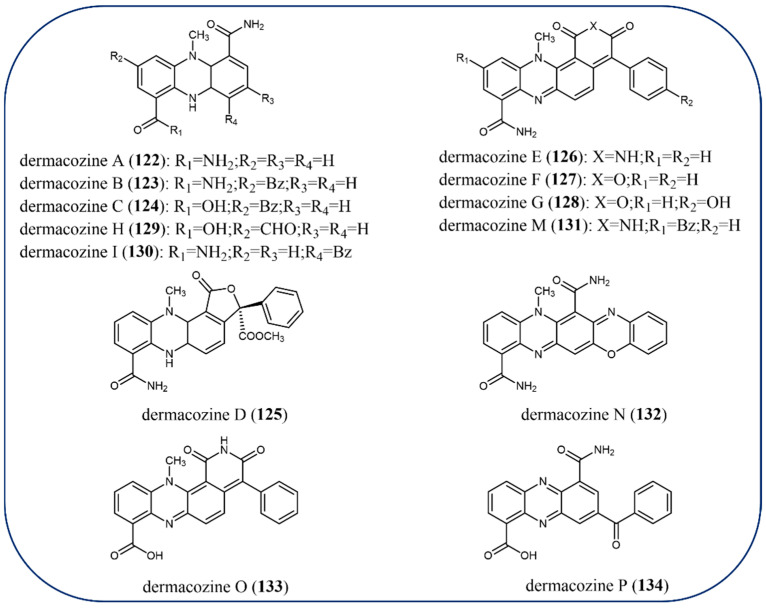
The chemical structures of dermacozines.

A series of new phenazine-type pigments dermacozines A–J (**122**–**130**, **101**) and dermacozines M–P (**131**–**134**) were discovered in the fermentation media of *Dermacoccus abyssi* sp. strains isolated from Mariana Trench sediment (Figure 13) [98]. Dermacozine N (**132**) is the first linear pentacyclic oxazinophenazine natural product discovered [99]. This chemical family of compounds showed a low cytotoxic effect against resistant cancer cell lines. Dermacozines F (**127**) and G (**128**) displayed the most potent cytotoxic activity. Dermacozine C (**124**) had the strongest radical scavenging ability.

**Figure 14:**
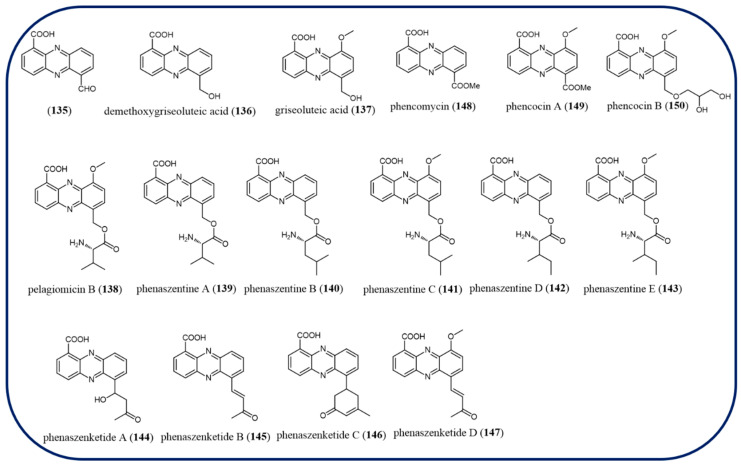
The chemical structures of compounds **135**–**147**.

A recently discovered phenazine intermediate, 6-formylphenazine-1-carboxylic acid (**135**) was thought to bridge the pathways encoded by the BGCs in nematodes and the symbiotic entomopathogenic bacterium *Xenorhabdus szentirmaii*. A variety of non-enzymatic and enzymatic reactions can be used to generate demethoxygriseoluteic acid (**136**), griseoluteic acid (**137**), and a number of derivatives such as pelagiomicin B (**138**), phenaszentines A–C (**139**–**141**), phenaszentines D (**142**), and E (**143**), phenaszenketides A–D (**144**–**147**), phencomycin (**148**), phencocins A (**149**), and B (**150**) (Figure 14) from **135** [100].

Table 1 lists different types of phenazine natural products with various biological activities in this review.

## 3. Phenazines of Therapeutic Interest

There have been many biological investigations related to natural and synthtic phenazines that demonstrate therapeutic value related to human health and disease. Phenazinomycin isolated from *Streptomyces* sp. WK-2057 bears a cyclic terpenoid appendage. The compound shows moderate antibacterial activity against *Staphylococcus aureus* and direct cytotoxic activities against HeLa S3, P388, and P388 doxorubicin-resistant cells. In addition, phenazinomycin demonstrated antitumor activity against experimental murine tumors (Sarcoma 180 cells) in vivo, resulting in a prolongation of survival time for tumor-bearing mice (up to 140%) [101]. Iodinin (**11**) demonstrated that hypoxia-selective antileukemia activity (EC_50_ = 2.0 μM against normoxic cells, EC_50_ = 0.79 μM against hypoxic cells) has the potential to act on malignant cells in hypoxic bone marrow of AML patients [46]. The halogenated phenazine natural product, 2-bromo-1-hydroxyphenazine (**78**) with an MIC = 6.25 μM, is significantly more potent than pyocyanin (MIC = 50 μM). Synthetic halogenated phenazine compounds HP 1 (MIC = 1.56 μM) and HP 29 (MIC = 0.08 μM) have potent antibacterial activity and are able to eradicate bacterial biofilms. HP 29 has demonstrated especially good efficacy in dorsal wound infection models in mice [102]. Clofazimine has been received FDA approval to treat lepromatous leprosy (caused by *Mycobacterium leprae*) in 1986 and is on the World Health Organization’s (WHO) model list of essential medicines [103,104]. XR11576 is an oral topoisomerase I and II inhibitor, and bis-phenazine XR5944 is a novel cytotoxic agent that intercalates into DNA and inhibits transcription [105,106]. These two compounds have been under phase I clinical testing. NC-182 is a novel antitumor compound with a benzo-[*a*]phenazine ring. It is a potent DNA intercalator with nearly the same binding ability as daunomycin; however, the mode of interaction of NC-182 with DNA depends on the concentration of the drug, where the intercalative and electrostatic bindings are dominant at low and high concentrations of the drug, respectively [107]. NC-190 has been shown to induce topoisomerase II-dependent DNA cleavage and DNA fragmentation. The compound demonstrates anticancer activity against multiple cancer cell lines in vitro and in vivo (tumor models in animals), and the activities against HL-60 cells in a dose-dependent manner were comparable to that of etoposide, which is a clinically used cancer therapy [108,109].

## 4. Conclusions

In this review, we summarized the research on the structural variety and biological activity of natural phenazine products over the last ten years. Over 150 phenazines with various structures were introduced. To summarize, phenazine products are prevalent in nature; however, currently known natural phenazines are only found in microbes. Nevertheless, there are diverse substituent groups that appear on the phenazine ring including carboxyl, hydroxyl, methyl, hydroxymethyl, carboxamide, terpenoid, glycosyl, halogen, sulfur, and some other complicated derivative groups. It can be concluded that phenazines from *Streptomyces* are deemed more complex, diversified, and fascinating based on the structures of phenazine natural products derived from different sources. The simple post-modified phenazines are mainly from Gram-negative bacteria, for instance, *Pseudomonas* and *Lysobacter antibioticus*. However, in Gram-positive bacteria strains, especially *Streptomyces*, not only simple phenazine derivatives but also complex phenazines can be obtained, pointing out the direction of the search for novel complex phenazine natural structures. Phenazines with diverse structures exhibit powerful anticancer and antibiotic activities. Hydroxylation and hydroxy-methylation of phenazines are very important for the antibiotic activity of phenazines. For example, PCA, 2-hydroxyphenazine, 1-methoxyphenazine, and 1-methoxyphenazine *N’* 10-oxide have great potential in preventing plant diseases and insect pests. Especially, PCA is widely used in southern China as a key fungicide to prevent rice sheath blight. Moreover, more than 100 companies in the world are now producing and selling PCA [110]. *P. chlororaphis* and *P. fluorescens*, which produce a wide range of bioactive phenazines, have been commercialized by companies from Europe (Cedomon BioAgri AB, Uppsala, Sweden) and the USA (AtEze, Turf Science Laboratories). PYO and PCN, which have potent antimicrobial activity, are typical representatives of *N*-methylated and carboxamidated phenazines, respectively. Halogenated phenazines can eradicate surface-attached biofilms with excellent potency, and phenazine di-*N*-oxides are an interesting class of antitumor agents that selectively kill the hypoxic cells found in solid tumors [49,111]. Most of the terpenoid and glycosylated phenazines do not have remarkable antibacterial activity. The biological activities of phenazines and the structure–activity relationship need further study.

Since PCA has been developed as a registered biopesticide, the application of phenazine derivatives will be promising in the field of agriculture pathogenic fungi control. The biosynthesis studies pave the way to create the green manufacturing approach of diverse phenazines. Although enzymes involved in phenazines, methylation, hydroxylation, glycosylation, and isoprenylation have been elucidated, and novel phenazine products have been artificially designed using these post-modification proteins, and there are still plenty of intriguing hidden biosynthetic pathways to be discovered, such as dimerization, halogenation, saphenic acid pathway derivatization, and methanophenazine biosynthesis. Therefore, there is a need to find pivotal enzyme reactions for the biosynthesis of phenazines with excellent activity. In most cases, the titer of naturally occurring products is insufficient for the application. Efforts should be undertaken to optimize the host strains in order to satisfy the demands of an industrial-scale production.

## Figures and Tables

**Table 1:** Biological activities of phenazines in this review.

Substance Class	Biological Activities	Compounds (Refs)
Simple derivatives	Antifungal activity	**1** [19,20,21,22,23,24,25,26]
	Antibacterial activity	**2** [28,29,30,31,32]
	Cytotoxic activity	**2** [30]
Phenazines with hydroxyl and methoxyl moieties	Antibacterial activity	**3**–**9**, **03**, **08** [34,35,36,37,38,39,41]
	Antifungal activity	**3**–**7**, **10** [34,35,36,37,38,39,42]
	Antitumor activity	**3**–**7** [34,35,36,37,38,39]
*N*-oxide phenazines	Antimicrobial activity	**11**, **12** [41,42,43]
	Antifungal activity	**19** [45,46]
	Cytotoxic activity	**11**, **12**, **17** [47,48,49,50]
*N*-methylated phenazines	Antibacterial activity	**20** [53,54,55]
	Antitumor activity	**5MPCA**, **20** [57]
	Antifungal activity	**20** [53,54,55]
Carboxamidated phenazines	Antifungal activity	**21**–**23** [61,62,64,65]
	Antitumor activity	**21** [63]
	Antibacterial activity	**22**, **23** [67]
Terpenoid phenazines	Antibacterial activity	**29**–**31**, **42**, **43** [68,73]
	Cytotoxic activity	**42**, **43** [73]
	Antifungal activity	**48**, **49** [74]
Glycosylated phenazines	Antibacterial activity	**44**–**46** [68], **50**–**52** [76]
	Cytotoxic activity	**54**, **55**, **59** [78]
Halogenated phenazines	Cytotoxic activity	**78**–**84** [83,84]
	Antimicrobial activity	**79**–**84** [84]
Saphenic acid derivatives	Antineuroinflammatory activity	**100** [88]
	Cytotoxic activity	**100** [88]
Phenazines with sulfur	Radical scavenging activity	**101** [91]
Special derivatives	Antitumor activity	**104**–**106** [93]
	Antifungal activity	**107**–**108**, **114**, **117**–**119** [42,95,96,97]
	Antibacterial activity	**109**, **114**, **117**–**119** [95,96,97]
	Cytotoxic activity	**117**–**119**, **122**–**134** [96,97,98,99]
	Radical scavenging activity	**124** [99]

## Data Availability

Not applicable.
